# Prohibitions in the meta-inflammatory response: a review

**DOI:** 10.3389/fmolb.2024.1322687

**Published:** 2024-05-15

**Authors:** Natalia Todosenko, Kristina Yurova, Maria Vulf, Olga Khaziakhmatova, Larisa Litvinova

**Affiliations:** ^1^ Center for Immunology and Cellular Biotechnology, Immanuel Kant Baltic Federal University, Kaliningrad, Russia; ^2^ Laboratory of Cellular and Microfluidic Technologies, Siberian State Medical University, Tomsk, Russia

**Keywords:** prohibitins, obesity, metabolic syndrome, cell metabolism, organelles

## Abstract

Prohibitins are the central regulatory element of cellular homeostasis, especially by modulating the response at different levels: Nucleus, mitochondria and membranes. Their localization and interaction with various proteins, homons, transcription and nuclear factors, and mtDNA indicate the globality and complexity of their pleiotropic properties, which remain to be investigated. A more detailed deciphering of cellular metabolism in relation to prohibitins under normal conditions and in various metabolic diseases will allow us to understand the precise role of prohibitins in the signaling cascades of PI3K/Akt, Raf/MAP/ERK, STAT3, p53, and others and to fathom their mutual influence. A valuable research perspective is to investigate the role of prohibitins in the molecular and cellular interactions between the two major players in the pathogenesis of obesity—adipocytes and macrophages - that form the basis of the meta-inflammatory response. Investigating the subtle intercellular communication and molecular cascades triggered in these cells will allow us to propose new therapeutic strategies to eliminate persistent inflammation, taking into account novel molecular genetic approaches to activate/inactivate prohibitins.

## 1 Introduction

Obesity is a global health problem (more than half of the US population will be obese by 2030) (Purdy and Shatzel, 2021) and is a predisposing factor for the development of dangerous components of the metabolic syndrome associated with a low-grade inflammatory state (non-infectious metainflammation) (Milić et al., 2014; Acosta-Martinez and Cabail, 2022; Fromenty and Roden, 2023). In obesity, the functional potential and properties of the cells of various tissues and organs are altered by molecular genetics and epigenetic mechanisms, leading to metabolic disorders and aging of the body (Wu et al., 2022).

Pathological alterations in cell metabolism and abnormal activation of innate immunity associated with excess fatty acids, their transport, accumulation of lipid droplets and disruption of interorganelle connections lead to reprogramming of nuclear DNA: expression of certain transcription factors and genes that activate the processes of cell aging, cell death and maintenance of the inflammatory response. The link between sterile metabolic inflammation in obesity and aging processes at the cellular and organismal level is emphasized (Lee, 2022; van de Vyver, 2023). An increase in the number of senescent cells, which differ in genotype and produce a secretory pro-inflammatory phenotype/senescence-associated phenotype (SASP), inhibit adipogenesis and induce the development of insulin resistance (IR) (Palmer et al., 2019). The inflammatory process is accompanied by an increased production of ROS leading to mitochondrial dysfunction: Swelling, changes in membrane potential, destructuring, release of mitochondrial DNA (mtDNA) (Todosenko et al., 2023b), disruption of the Krebs cycle and respiratory complexes, increase in oxidative stress. Cellular disorders in obesity and metabolic syndrome affect not only peripheral tissues (adipose tissue) but also the central nervous system (hypothalamus, hippocampus, blood-brain barrier, cerebrospinal fluid) (de Mello et al., 2018), which underlines the complex developmental mechanism and systemic manifestation of these pathologies.

Modern research points to an important role of proteins of the prohibitin family (PHBs) in cellular homeostasis. The discovery of PHBs dates back to 1989. Their name refers to their originally identified intranuclear function, the inhibition of cell proliferation (Jiang T. et al., 2022). PHBs are ubiquitously expressed (in high concentration in cells with high energy expenditure) (Signorile et al., 2019). These proteins are localized in the cell membrane, mitochondria, nucleus and cytoplasm (Thuaud et al., 2013; Mattox et al., 2021; Matthews et al., 2023) and exhibit a tissue-specific functional potential that depends on the conditions created (for pathologies). The structural domains of PHBs facilitate their intracellular movement and provide signaling communication between organelles (Alula et al., 2021). PHBs regulate cell activity via different mechanisms (MacVicar and Langer, 2016). The anti-inflammatory potential of PHBs has been demonstrated and targets the modulation of signaling pathways in innate immune cells (Matthews et al., 2023), the signaling of IgM receptors in B cells (von Wenserski et al., 2021) and the maturation of T cells (Mishra and Nyomba, 2019). In addition, the expression of PHBs is significantly reduced in senescent cells, which leads to increased lipid levels and mitochondrial hyperplasia and increases oxidative stress (Yan et al., 2020). Multivariate genome-wide analyzes confirmed that the expression of PHBs genes is a key factor in the aging of organisms (Rosoff et al., 2023). At the same time, high expression of PHBs in cancer cells contributes to their escape from apoptosis and development of chemoresistance, confirming their role in cell cycle regulation in health and disease (Belser and Walker, 2021). Of particular interest in this context is the role of PHBs and their complexes in stabilizing/maintaining the integrity and regulation of the mitochondrial genome (He et al., 2022). The involvement of PHBs in the processes of adipogenesis and lipid metabolism, the dysregulation of which is observed in metabolic diseases (Ande et al., 2017; Jung et al., 2020).

Despite the available (rather limited) data on the biology and effects of PHBs in the regulation of cellular processes under normal conditions/conditions of various pathologies, many aspects of their molecular action remain unexplored, which does not allow us to determine their comprehensive role in complex communicative intercellular connections and the development of the inflammatory response in obesity and components of the metabolic syndrome. Considering that most scientific work has been performed on yeast, mice and cell cultures, a comprehensive understanding of the role of prohibitins in metabolic diseases is far from being achieved. However, the research results indicate that prohibitins could be a promising therapeutic target in the fight against serious, socially significant diseases.

The aim of the review is to describe the molecular genetic role of prohibitins in the development of the inflammatory process associated with cell aging in obesity and metabolic syndrome.

## 2 Functions of prohibitins in relation to their localization in obesity (metabolic syndrome)

The prohibitin family comprises several proteins, of which Prohibitin 1 (PHB1) and Prohibitin 2 (PHB2) are the best studied. PHB1 and PHB2 have a molecular weight of 31 kDa and 37 kDa (Zhang et al., 2024) or 32 and 34 kDa (Signorile et al., 2019).

The PHB1 gene has been assigned to chromosomal locus 17q12-q21, near genes responsible for the deposition of adipose tissue and genes that regulate eating behavior and sex hormone levels (Mishra et al., 2010). The PHB2 gene is located on chromosome 12p13 (Zhang et al., 2024) next to the AdipoR2 gene (12p13.33), which codes for the adiponectin receptor (adipose tissue hormone) and is associated with obesity and the hypothalamic satiety response (Mora-García et al., 2017).

Both PHBs have a similar structure and consist of a C-terminal helical domain, which is involved in protein-protein interactions (with each other and with transcriptional regulators), and an N-terminal transmembrane domain, which is an evolutionarily conserved zapertin domain. Human PHB2 has a non-cleavable mitochondrial target sequence at the N-terminus and a nuclear localization signal at the C-terminus (Kasashima et al., 2006). At the same time, PHB1 differs in structure from PHB2 and its mitochondrial translocation is ensured by Akt-dependent phosphorylation of Thr258 (Jiang et al., 2015).

In the mitochondrial membrane, PHB1 and PHB2 together form a ring-shaped structure—a complex of 12–20 PHB heterodimers with a molecular weight of 1 MDa (Signorile et al., 2019).

PHB1 and PHB2 have conserved phosphorylation sites (Ande et al., 2017). At the same time, several post-translational modifications of PHB1, including palmitoylation, ubiquitination, phosphorylation and cysteine oxidation (Ande et al., 2017), as well as other stimuli (Fusaro et al., 2003; Lee et al., 2010; Dong et al., 2013), cause its cellular movement between organs (Ande and Mishra, 2010).

PHB1 and PHB2 have multifunctional potential related to subcellular localization, posttranslational modifications and the interactome (Thuaud et al., 2013; Peng et al., 2015).

### 2.1 Сytoplasmic membrane

**FIGURE 1:**
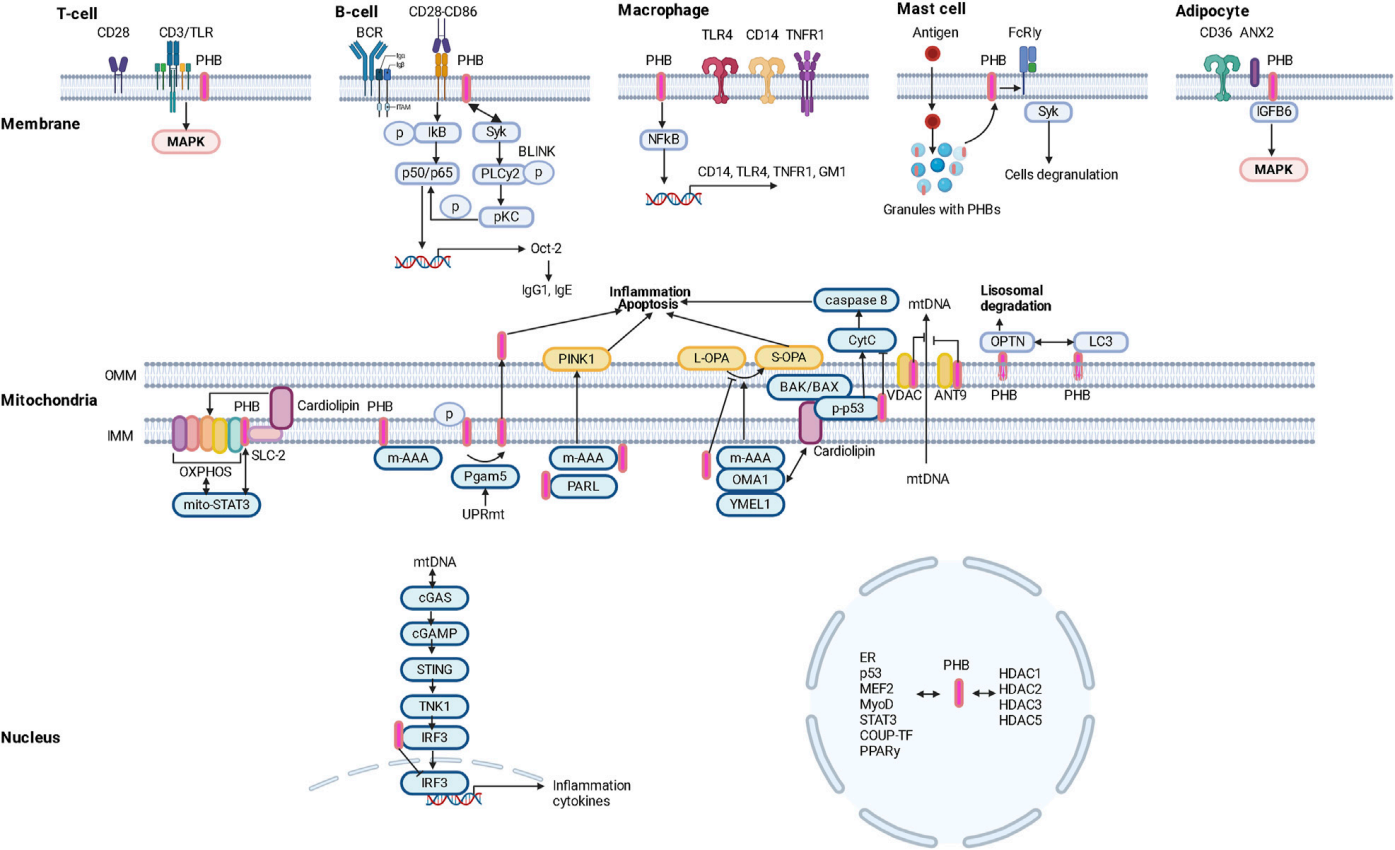
Intracellular localization and functional potential of prohibitins (PHBs).

Membrane PHBs (Zhang et al., 2022) regulate the formation/composition of lipid rafts, fatty acid transport and the development of the inflammatory response (Figure 1).

#### 2.1.1 Lymphocytes

Colocalization of PHBs with the CD3 molecule of the TCR complex (link to MAPK signaling) and the BCR may determine its functions in the regulation of immune responses. PHBs modulate CD86-dependent phosphorylation of IκBα and thus mediate NF-κB p50/p65 activation in B cells. PHBs also interact with the tyrosine kinase Syk, which induces the phosphorylation of PLCy2 and the subsequent activation of PKCα/βII as well as the phosphorylation of p65 in a BLNK-mediated manner. NF-kB p50/p65 then initiates transcription of the Oct-2 gene and increases the production of IgG1 (as well as IgE) (Lucas et al., 2013; Matthews et al., 2023).

Obesity in animals has been found to lead to a reduced immune response to antiviral vaccination, which can be explained not only by impaired function of the macrophage component of immunity (Cho et al., 2016), but also by reduced expression of the membrane protein PHBs in T and B lymphocytes (Figure 1).

#### 2.1.2 Monocytes/macrophages

PHB1 and PHB2 are a coherent system. PHB2 in macrophages regulates the production of proinflammatory mediators (cytokines/chemokines) by modulating the expression of CD14, TNFR1, TLR4 and the lipid raft marker ganglioside GM1 (Matthews et al., 2023). Suppression of PHB2 resulted in decreased PHB1 expression and attenuated NF-kB activation with subsequent changes in cyto/chemokine production associated with decreased surface localization of TNFR1/CD14/TLR4 and GM1 and impaired lipid raft formation/molecular restructuring of the plasma membrane (Matthews et al., 2023) (Figure 1).

Thus, the earlier study suggests a link between PHB and TLR4 expression. This could be a promising area of research as PHB may regulate TLR4-mediated energy homeostasis in the hypothalamus, which is activated by HFD and leads to inflammation, weight gain, insulin and leptin resistance via the IKKβ/NF-kB signaling cascade and endoplasmic stress. Reticulum (the JUN signaling pathway is activated in adipose tissue, liver and muscle) (Ullah et al., 2021).

Obesity and type 2 diabetes mellitus are characterized by changes in the population composition of monocytes and macrophages that lead to immune dysfunction (Todosenko et al., 2023a), in particular by a decrease in the expression of the CD86 marker, which is characteristic of HLA-DR-positive macrophages with pro-inflammatory potential (M1) (Valtierra-Alvarado et al., 2020). It is known that PHB is localized in close proximity to the surface receptor CD86 and promotes CD28-CD86 signaling (Valtierra-Alvarado et al., 2020). Since PHB are surface receptors of monocytes/macrophages, they could influence the development of local and systemic inflammatory responses in metabolic diseases (Zhang et al., 2022) and promote enhanced PHB-mediated invasion of pathogenic microorganisms (viruses and bacteria) into cells (Liu et al., 2016) (Figure 1).

#### 2.1.3 Mast cells

PHB1 and PHB2 are located on the cell membrane of mast cells and promote their activation/degranulation (Zhang et al., 2022; Matthews et al., 2023).

A predominant localization of PHB1 in granules of mast cells and its translocation into lipid rafts of the plasma membrane against a background of antigenic stimulation was found. Lyk-phosphorylation of PHB1 on tyrosine was stimulated by the action of antigens. Palmitylated PHB1 forms a membrane complex with the IgE receptor FcεRIγ and the non-receptor tyrosine kinase Syk, whose activation leads to cytokine production and cell degranulation (Yurugi and Rajalingam, 2013) (Figure 1).

The role of mast cells stimulated by non-allergic triggers (neuropeptides, cytokines, adipokines) in inflammatory processes during the progression of obesity and type 2 diabetes mellitus (Theoharides et al., 2011; Kumar et al., 2019) has been established. Adipokines induce mast cell migration via an ERK1/2-mediated mechanism, leptin induces secretion of histamine, cysteinyl leukories and CCL2 expression; adiponenctin induces IL-10 production by activating the PI3K/p38 signaling pathway (Milling, 2019). This is consistent with the increased number of mast cells in adipose tissue and their role in the recruitment/activation of proinflammatory immune cells (triggered by mast cell degranulation and cytokine production) and fibrosis/remodeling of adipose tissue in obesity (Żelechowska et al., 2018).

#### 2.1.4 Adipocytes

In addition, the surface expression of PHB1 is a characteristic surface marker of endothelial cells in the white adipose tissue (WAT) of mice and humans (Kolonin et al., 2004) (Figure 1).

The important role of high expression/depletion of the cellular glycoprotein CD36 and its free form sCD36 in peripheral blood and dysfunction of CD36 in the hypothalamic region of the brain (Le Foll et al., 2015), which is associated with taste sensitivity (Li et al., 2022b, p. 36) in the development of obesity, hyperinsulinism and metabolic syndrome (Karunakaran et al., 2021) has been demonstrated (Le Foll, 2019). In particular, CD36 (Valtierra-Alvarado et al., 2020) is a marker for metabolically activated macrophages (MMe), which are involved in the development of obesity. In addition, the uptake and metabolism of long-chain fatty acids in adipocytes and endothelial cells depend on the CD36/ANX2/PHB1 membrane complex. Superficial expression of PHBS in adipocytes and cardiomyocytes has been shown to regulate the synthesis/metabolism of lipids through CD36-dependent transport of fatty acids and oxidation of carnitine palmitransferaza 1B and inhibition of pyruvate carboxilase (Yanran et al., 2022; Matthews et al., 2023). Post-translational modification of CD36 in conjunction with S-acylation of ANX2 and PHB1 in adipocytes allows it to dissociate from ANX2/PHB1 and translocate to and from lipid droplets under lipolytic conditions (Daquinag et al., 2021) (Figure 1).

PHB2 in the plasma membrane directly contacts insulin-like growth factor binding protein 6 (IGFBP 6) and regulates cancer cell migration by activating MAP kinases (Bach, 2015), IGF1R (GALNT14/PHB2/IGF1R) (Fu et al., 2013; Chu et al., 2022).

At the same time, insulin-like growth factors and insulin-like growth factor binding proteins (IGFBPs) regulate cell proliferation/differentiation and may play a crucial role in the development of obesity and IR (Minchenko et al., 2019; Czogała et al., 2021). Childhood obesity has been associated with decreased IGFBP6 levels, which warrants further investigation (Czogała et al., 2021).

### 2.2 Mitochondria

**FIGURE 2:**
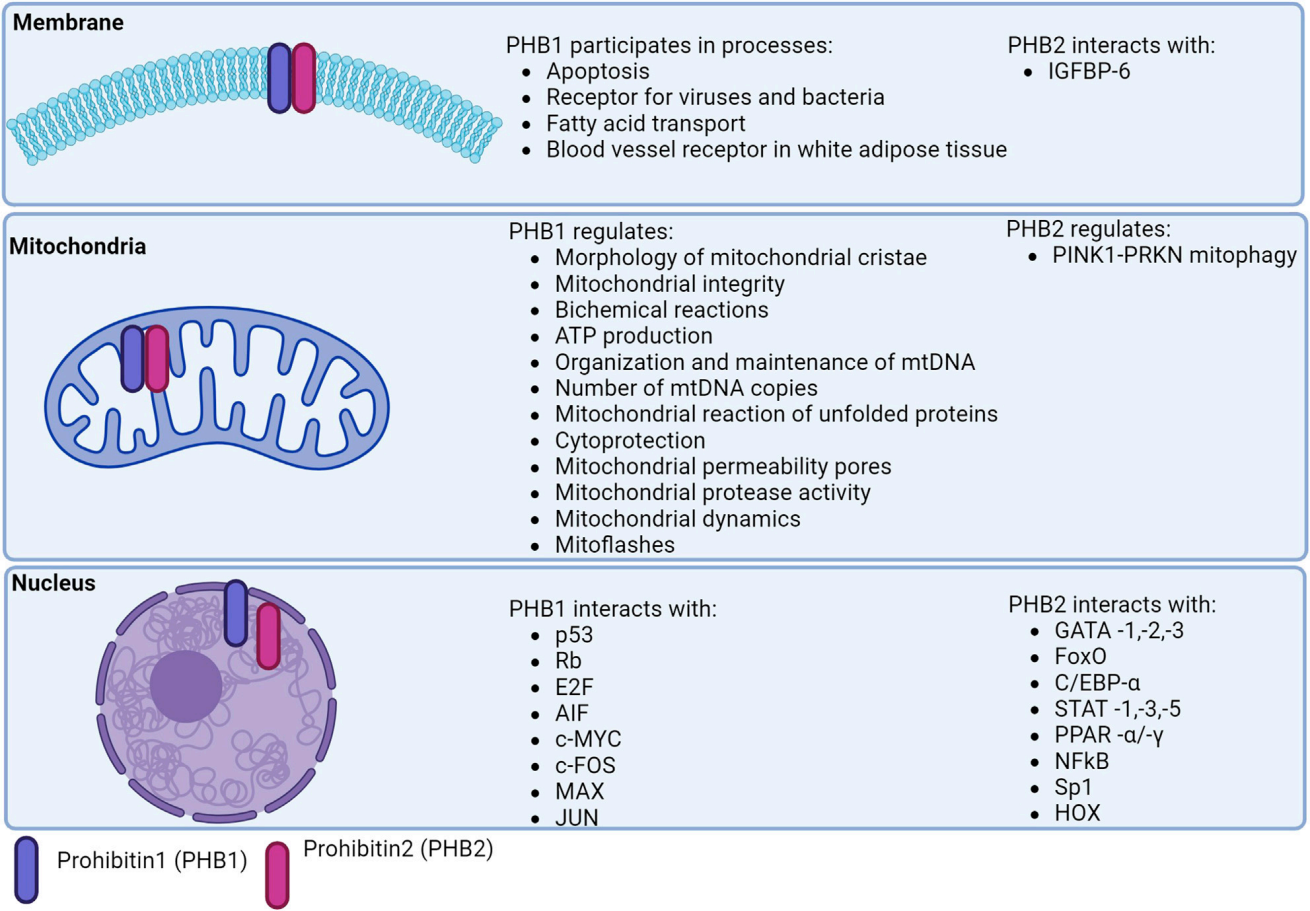
Localization of prohibitins in the cell.

Mitochondria maintain the balance between pro- and anti-apoptotic factors by producing ROS via the oxidative phosphorylation system (OXPHOS). The OXPHOS system consists of five multimeric protein complexes that are embedded in the inner mitochondrial membrane (IMM) and are under the genetic control of the mitochondrial nuclear genome. The mitochondrial DNA (mtDNA) encodes 37 gene products, including 13 OXPHOS polypeptides, which are transcribed/translated locally in the mitochondria (Tang et al., 2020) (Figure 2).

#### 2.2.1 Oxidative phosphorylation

PHBs are involved in the degradation of subunits of the respiratory chain, the assembly and activity of OXPHOS, mitochondrial biogenesis, apoptosis and mitophagy (Signorile et al., 2019).

PHBs physically interact with subunits of the OXPHOS complex (Bourges et al., 2004) and maintain its stability. Deletion of somatin-like inner mitochondrial membrane (IMM)-associated protein 2 (SLP-2), which interacts with PHBs, resulted in proteolysis of PHBs and subunits of the OXPHOS complex (I, IV) in the HeLa cell line (Da Cruz et al., 2008). It is suggested that when SLP-2 forms a complex with PHB, it binds the mitochondrial phospholipid cardiolipin and regulates the stability of the respiratory supercomplex (Anderson et al., 2018). SLP-2 has been reported to regulate mitochondrial translation through association with mitoribosomes (Mitsopoulos et al., 2017). When the expression of PHBs was inhibited, a decrease in mitochondrial protein synthesis was observed in mouse embryonic fibroblasts (He et al., 2012) (Figure 1).

Sphingosine kinase 2 (SphK2) is produced by mitochondria and is involved in the metabolism of ceramides, from which the lipid mediator sphingosine-1-phosphate (S1P) is ultimately formed, which activates the immune function of lymphocytes (Vaena et al., 2021). SphK2 interacts with PHB2 and regulates the lateral mitochondria. The contact of SphK2 with PHB2 brought the enzyme SphK2 closer to its mitochondrial target. In addition, PHB2 co-immunoprecipitates with complex IV subunit I (COX) of the OXPHOS-terminal complex. At the same time, depletion of SphK2 or PHB2 was accompanied by a decrease in the abundance of respiratory complexes II and IV and a less pronounced change in complex I in cardiomyocytes, MCF7, HeLa cell lines and PC12 neurons. These data suggest a role for PHB2 in the proper assembly of the OXPHOS complex and modulation of mitochondrial respiratory function (Strub et al., 2011).

Immune responses, growth, and the response to cellular stress are associated with activation of the transcription factor STAT3, which translocates to the mitochondria via the outer mitochondrial membrane protein Tom20 along with heat shock protein 22 (mitoSTAT3) and regulates mitochondrial respiration when it interacts with the I and II complexes of OXPHOS and the GRIM-19 protein (Tammineni et al., 2013). PHB1 has been found to interact with mitoSTAT3 in enterocytes to prevent inflammation-induced mitochondrial dysfunction (Han et al., 2014).

The mitochondrial PHB complex forms a supercomplex with the m-AAA protease (Bassi et al., 2021) and is responsible for the proteolysis of IMM proteins.

#### 2.2.2 Mitochondrial biogenesis

At the same time, the number of mtDNA copies is considered an indicator of the functional potential of mitochondria and the degree of oxidative stress, which decreases with metabolic diseases and aging of the body (Li M. et al., 2023). The mitochondrial transcription factor A (TFAM) controls the mtDNA copy number. However, high expression of TFAM leads to deficient oxidative phosphorylation (and postnatal mortality) in mice (Bonekamp et al., 2021).

Mitochondrial biogenesis increases with cell differentiation. Preadipocyte differentiation is associated with increased expression of PHBs proteins. Knockout of PHBs reduced the expression of adipogenic markers, lipid accumulation and low mitochondrial content. Mice overexpressing PHB1 developed obesity associated with increased mitochondrial biogenesis (Ande et al., 2014) in the presence of high copy numbers of mtDNA and mitochondrial proteins of transcription factors regulating mitochondrial biogenesis and nuclear genes encoding protein structures of the OXPHOS complex (PGC-1α, NRF2, OPA1, DRP1, TFAM) (Ande et al., 2014). PHB1 stabilizes TFAM in a chaperone-like manner and induces PGC-1α expression by regulating mtDNA copy number. Inhibition of PHBs led to a decrease in mtDNA copy number and induced cell apoptosis in HeLa cells (Kasashima et al., 2006) (Figure 1).

#### 2.2.3 Unfolded protein response

Cellular stress leading to mtDNA depletion, loss of mitochondrial membrane potential, imbalance between proteins encoded in the nucleus and mitochondria, and accumulation of unfolded proteins in the mitochondria activates signals that trigger the transcriptional program of the nucleus to restore mitochondria and induce expression of mitochondrial proteases and chaperones that control folding assembly. protein degradation—response to unfolded proteins (UPRmt) (Naresh and Haynes, 2019; Oyang et al., 2022). An early indicator for the activation of UPRmt is the correct synthesis of the sphingolipid S1P, which is mediated by the sphingosine kinase SPHK-1 (Kim and Sieburth, 2018). In this case, as already mentioned, SPHK-1 interacts with PHB.

The UPRmt response is accompanied by the activation of the serine/threonine protein phosphatase Pgam5, which induces the dephosphorylation of PHB2 Ser39 (in cardiac muscle and liver cells) and the translocation of PHB2 from the mitochondria to the cytoplasm, triggering apoptosis associated with inflammation (Cai et al., 2023, 2023). However, loss of Pgam5 inhibited inflammation by preventing the dephosphorylation of Bax mediated by the release of mtDNA (Li J. et al., 2023) (Figure 1).

PHBs (Oyang et al., 2022) are directly involved in the UPRmt process.

#### 2.2.4 Removal/repair of damaged mtDNA

Oxidative stress leads to mitochondrial dysfunction by disrupting the structure of mtDNA, its fragmentation/accumulation and exit from mitochondria, and triggering mitophagy via three different pathways: canonical PINK1-PRKN, non-canonical ATAD3 and FUNDC1 (Liao et al., 2022), which attempt to maintain mitochondrial functionality (Figure 1).

##### 2.2.4.1 PINK1-PRKN–mediated mitophagy

Mitophagy is a selective form of macroautophagy that aims to degrade dysfunctional/excess mitochondria by autophagolysosomes. The control of mitophagy is related to the activity of mitochondrial cargo receptors, which include PHB2 (Poole and Macleod, 2021). Mitochondrial quality control is related to PTEN-induced kinase 1 (PINK1), which is localized in the mitochondrial membrane, and cytosolic Parkin RBR E3 ubiquintin protein ligase (PRKN/Parkin). PINK1 is processed and degraded by mitochondrial proteases (m-AAA, i-AAA, PARL). Mitochondrial damage leads to disruption of PINK1 proteolysis and accumulation of PINK1 involving PRKN, which ubiquitinates outer mitochondrial membrane proteins and leads to autophagosome formation and organelle elimination.

PHB2 plays a crucial role in PINK1/PRKN mitophagy. Proteasome-mediated rupture of the mitochondrial membrane leads to the interaction of PHB2 with MAP1LC3B/LC3 (Wei et al., 2017), proteins that envelop phagophores and interact with damaged membranes to lysosomally degrade them (Stolz et al., 2014). PHB2 contains an LIR domain consisting of amino acids 121–124 (YQRL) located in the space between the IMM and the outer mitochondrial membrane (OMM). The LIR domain of PHB2 binds directly to LC3 and regulates mitophagy (Wei et al., 2017; Yan et al., 2020). In addition, PHB2 can bind to the autophagy receptor optineurin (OPTN) via the LIR domain, which is translocated to the mitochondria under the influence of PINK1-mediated phosphorylation of PRKN, leading to the recruitment of LC3 and other autophagic factors (ULK1, DFCP1, WIPI1) (Zhang et al., 2024). Alternatively, PHB2 may interact with the UBAN domain of OPTN via ubiquintin chains to regulate autophagy (Zhang et al., 2024). In this case, PHB2 may play a dual role in mitophagy: as a receptor and as a protein to recruit autophagosomes (Figure 1).

PHB2 deficiency inhibited mitochondrial recruitment of PRKN and blocked mitophagy. Accumulation of unfolded proteins in the mitochondrial matrix induces PRKN recruitment independent of mitochondrial membrane potential (Jin and Youle, 2013). Furthermore, depletion of PHB2 inhibited mitochondrial accumulation of unfolded protein induced by mitochondrial recruitment of PRKN. PHB2 is thought to inhibit the rupture of the outer mitochondrial membrane upon its depolarization (Yan et al., 2020).

In addition, activated PRKN induces rupture of the OMM via the RING2 domain and binds directly to PHB2, promotes ubiquintylation of the K33/K142 site of PHB2 and induces the interaction of PHB2 with LC3 (Zhang et al., 2024) (Figure 1).

PARL leads to cleavage of PGAM5 by the serine/threonine protein phosphatase, which stabilizes PINK1(Sekine et al., 2012). PHB2 stabilizes PINK1 in a PARL-mediated manner and regulates PINK1/PRKN mitophagy independently of interaction with MAP1LC3B. In addition, PHB1/2 interacts with PARL, which is associated with mitochondrial membrane depolarization. Upon membrane depolarization, PHB2 preferentially interacts with PARL rather than PGAM5, triggering mitophagy through PGAM5 and supporting a role for PARL-PGAM5 in PHB2-mediated stabilization of PINK1(Yan et al., 2020).

It was found that PHB2-associated mitophagy could be related to the activity of the Golgi apparatus, confirming the interaction of PHB2 with Golgi phosphoprotein 3, which triggers PHB2-LC3 signaling (Zhang et al., 2024).

Of interest is the role of PHB2 in inhibiting the conversion of LC3I (microtubule-associated light chain) to LC3II. Some authors refer to mitophagy associated with PHB2 activation as a novel type of process (Jiang M. et al., 2022). The basis of PHB2-induced mitophagy is the ability of PHB2 to stabilize the inner mitochondrial membrane protease PARL by preventing the degradation of PGAM5, which, when intact, stabilizes PINK1 and recruits PRKN and other receptors to promote mitophagy (Yan et al., 2020) (Figure 2).

##### 2.2.4.2 ATAD3–mediated mitophagy

Damage to mtDNA induces mitochondrial membrane remodeling and recruitment of endosomes to mitochondrial nucleoids. Non-cononic, endosome-dependent mitophagy promotes the removal of mtDNA fragments and is associated with the ATAD3 (mitochondrial ATPase)-SAMM50 axis (which controls mitochondrial cristae architecture), in which SAMM50 regulates BAK clustering and nucleoid release and facilitates their transfer to endosomes which mature with the participation of the VPS35 protein (Sen et al., 2022; Sen et al., 2023). SAMM50 interacts with VPS35 in the outer mitochondrial membrane and sequesters and eliminates mtDNA, which prevents the development of an enhanced immune response (Sen et al., 2022). ATAD3 is a heterodimerized mitochondrial receptor complex - ATAD3A and ATAD3B (a form expressed only in primates), and is also an important component of nucleoid proteins such as MTERF2, SSBP1, POLG, LONP1 (Liao et al., 2022). ATAD3A binds directly to mtDNA and is involved in the regulation of mtDNA transcription (Liao et al., 2022). In addition, ATAD3A regulates cristae together with OPA1, Yme1L, MICOS, SAM50(Peralta et al., 2018) (Figure 1).

In the rest of the ATAD3A-SATAD3B complex, mitochondria are localized in the intermembrane space, but mtDNA damage is weakened by heterodimerism of the complex, leading to exposure of LIR-containing ATAD3B-CONTRAN on the outer membrane of mitochondria and recruitment of LC3 with subsequent activation of Pink1 (Shu et al., 2021; Liao et al., 2022) (Figure 1).

At the same time, ATAD3A interacts with the components of the mitochondrial channels Tom40 and Tim23 and thus ensures the processing of the PINK1 protein. Depletion of ATAD3A led to an accumulation of PINK1 and activation of mitophagy (Jin et al., 2018). However, according to other data, induced depletion of ATAD3A in Huh7 cells resulted in suppression of PINK1 and blockage of mitophagy in the presence of a high-calorie diet and cholesterol overload, mediating the development of NAFLD (Chen et al., 2022).

##### 2.2.4.3 Mitophagy mediated by other mitochondrial receptors

Mitophagy is also triggered by receptor proteins such as FUNDC1 (FUN14 domain containing 1), NIX/BNIP3L (BCL2 interacting protein 3), BNIP3 and PHB2. In addition to PHB2, the other proteins also contain an LC3-binding motif, the LIR domain, which interacts with LC3 and activates mitophagy (Liao et al., 2022) (Figure 1).

FUNDC1 reduces autophosphorylation during hypoxia and interacts with LC3B to activate mitophagy. Loss of FUNDC1 disrupts the mitochondrial quality system and leads to metabolic disorders in mice. Loss of FUNDC1 in hepatocytes is associated with the release of mtDNA and the development of an inflammatory response. NIX and BNIP3 are activated by hypoxia and mtDNA damage to remove dysfunctional mitochondria. PHB2 triggers mitophagy upon stress and binds to LC3 via the LIR domain, which plays an important role in the removal of damaged mtDNA (Liao et al., 2022).

#### 2.2.5 Structure of the mitochondrial membranes

Mitochondria are characterized by a high frequency of fusions (mitofusins Mfn1/2, OPA1) and fissions (DRP1, Fis1) involving inner and outer membrane proteins (Pernas and Scorrano, 2016).

MiR-361-mediated suppression of PHB1 in cardiomyocytes led to mitochondrial fission, fragmentation and apoptosis, accompanied by the development of myocardial infarction (Wang et al., 2015). This is mediated by the stabilizing effect of the mitochondrial PHBs complex on the OPA1 protein, which regulates cristae structure, apoptosis and mitochondrial fusion. OPA1 is converted from the long L-form to the cleaved short S-form under the influence of various proteases (m-AAA, OMA1, YMEL1) (Zhang et al., 2024). PHB has been shown to interact with and inhibit the m-AAA protease, thereby preventing OPA1 (S-OPA1) processing and apoptosis. However, a lack of PHBs may stabilize the active form of the OMA1 protease and promote the cleavage of L-OPA1 (long form to short form) (Zhang et al., 2014) (Figure 1).

PHBs affect the maturation of the phospholipid component of the inner mitochondrial membrane involved in OPA1-dependent mitochondrial fusion, cardiolipin (Richter-Dennerlein et al., 2014). In addition, PHB interacts with cardiolipin and ensures the stability of the CI subunit of the respiratory supercomplex under conditions of oxidative stress (Zhang et al., 2024). It is likely that after Mfn-mediated fusion of the outer mitochondrial membrane, fusion of the inner membrane occurs with coordinated involvement of cardiolipin and the L-/S-forms of OPA1(Ge et al., 2020).

Liver cells from Phb2 knockout mice (Hep) showed excessive L-OPA1 degradation, mitochondrial fragmentation and increased apoptosis in the presence of glucose overproduction (Li et al., 2019).

#### 2.2.6 Apoptosis

Mitochondrial apoptosis is associated with the release of cytochrome C and the remodeling of mitochondrial cristae.

Overexpression of PHB1 in cardiomyocytes prevented the induced release of cytochrome C and decreased Bcl2 protein levels (Muraguchi et al., 2010). PHB has been shown to attenuate high glucose concentration-induced apoptosis of HRCEC cells by inhibiting caspase-3 and PARP (Zhang and He, 2022) (Figure 1).

Cytochrome C release is associated with changes in mitochondrial membrane phospholipid composition and scaffold function, which is dependent on PHB. Cardiolipin is involved in the release of cytochrome C and ensures the activation of caspase-8 on the surface of the mitochondria (Jalmar et al., 2013). PHB interact with the cardiolipin regulatory protein DNAJC19 and promote the acylation of monolysocardiolipin, resulting in cardiolipin and the lipid structure of mitochondrial membranes (Richter-Dennerlein et al., 2014). The absence of mitochondrial DNAJC19 resulted in impaired cristae morphology (Richter-Dennerlein et al., 2014), similar to depletion of PHB2 in MEF cells (Merkwirth et al., 2008) (Figure 1).

OPA1 is involved in cristae remodeling in the early stages of apoptosis. OPA1 oligomers are destabilized in the initial phase of apoptosis and induce cristae opening and cytochrome C release. It is suggested that the PHBs complex may be integrated into the vertical axis of mitochondrial cristae, thus ensuring their stabilization and an additional anti-diffusion barrier (Osman et al., 2009). In addition, the PHBs complex can stabilize the binding of the mitochondrial protease OMA1 and cardiolipin, inhibiting the formation of the S-form of OPA1 (Zhang et al., 2014), without cristae destruction and cytochrome C release, thereby blocking caspase-9-induced apoptosis (Zhang et al., 2024). BAK/BAX proteins interact with cardiolipin and induce mitochondrial outer mitochondrial membrane permeabilization (MOMP) during apoptosis (Dai et al., 2023) and activate OMA1 (Zhang et al., 2024). In the MOMP process, oligomerization of the BAK protein plays an important role, which is mediated by the interaction with the p-p53 (ser15) protein and precedes the activation of OMA1 and the processing of L-OPA1(Kong et al., 2022). At the same time, the formation of p-p53 (ser15)-PHB complexes in the mitochondria regulates the process of apoptosis (Kong et al., 2022). PHB acts as a chaperone protein for p53 (Thuaud et al., 2013). Stimulated apoptosis mediates the nuclear translocation of p53-PHB complexes (Li et al., 2015) by altering BAX transcription (Liu et al., 2012). The association of PHB with p53 is also observed in the nucleus, suggesting a reciprocal modulation of PHB expression by p53 (Kong et al., 2022). It is suggested that there is a link between the BAK1-BAX and OPA1-PHB2-OMA1 signaling cascades during cell apoptosis (Zhang et al., 2024), where p53 may be the link (Liu et al., 2012). Epigenetic factors regulate PHB activity during cell apoptosis. Oxidative stress activates miR-23A, which upregulates the transcription of p53 and induces the expression of miR-128, which targets PHB and leads to apoptosis (Li et al., 2015).

PHB2 modulates the stability of the anti-apoptotic HCLS1-associated protein X-1 (HAX1). A reduction in mitochondrial PHB2 leads to a reduction in HAX1 and an induced loss of mitochondrial integrity and apoptosis mediated by the activation of caspase-9/-3 (Kasashima et al., 2006). Reduction of PHB2 or OPA1 levels stimulated proteolysis of HAX1. It is likely that PHB2 also forms complexes with proteins that regulate mitochondrial permeability during apoptosis–VDAC, ANT3.

PHB1 modulates the nuclear expression of genes encoding proteins involved in apoptosis. The anti-apoptotic role of the mitochondrial PHB complex is supported by its high expression in tumor cells mediating resistance to chemotherapy (Tortelli et al., 2017; Signorile et al., 2019) (Figure 2).

## 3 Molecular mechanism of mtDNA release from mitochondria that triggers an inflammatory response (damage/alteration of mitochondrial membrane potential)

Mitochondrial apoptosis is regulated by the B-cell lymphoma 2 (BCL2) protein family, BCL-associated protein X (BAX) and the BCL2 antagonist Killer 1 (BAK). When BAX/BAK is activated, the outer mitochondrial membrane becomes permeable and releases proteins from the intermembrane space (cytochrome C) into the cytoplasm. A number of molecular models of BAX/BAK-based protein pores of different sizes with different permeabilization of the outer mitochondrial membrane have been proposed, the important role of which is the release of mtDNA in the context of inflammatory cell death (Liu et al., 2022). Oligomeric proteins of the voltage-dependent anion channel (VDAC) on the outer mitochondrial membrane contact BAX/BAK and simultaneously serve as receptors for the association of BAX with the outer membrane and as a platform for the retrotranslocation of BAX (Wolf et al., 2022). Alteration of mitochondrial membrane permeability induces mtDNA leakage through VDAC, BCL2, macropores, BCL2-associated regulators of apoptosis (BAX/BAK) (McArthur et al., 2018). PHBs have been found to be involved in the oligomerization of mitochondrial BAK proteins (Kong et al., 2022).

The release of short fragments of mtDNA from mitochondria in cells lacking endonuclease G is mediated by VDAC oligomers (Kim et al., 2019). BAX/BAK macropores release mtDNA during apoptosis (Rongvaux et al., 2014). In addition, several proteins induce the opening of mPTP at the inner mitochondrial membrane, leading to the release of mtDNA and the development of an inflammatory response (Wang et al., 2021). Components of mPTP are adenine nucleotide translocase (ANT), ATP synthase F1F0 and Optic Papplegia Protein 7 (SPG7), which regulate ion transport across the mitochondrial membrane. SPG7 forms a complex with m-AAA and AFG3-like protein 2 (AFG3L2) and also interacts with the mPTP component cyclophilin D (CYPD).

PHB1 negatively regulates m-AAA activity and presumably promotes mtDNA release by regulating SPG7-CYPD-dependent mPTP opening and activating the cGAS-STING signaling pathway and inducing the formation of an inflammasome with the production of the mature peptides IL-1β and IL-18 (Liu et al., 2022) and the production of IFN.

### 3.1 mtDNA-induced inflammatory response

#### 3.1.1 cGAS-STING

Free (cytoplasmic) mtDNA binds to the nucleic acid pattern recognition receptor (cyclic cyclic GMP-AMP synthase cGAS) and leads to the synthesis of CDN-2′,3′-cyclic guanosine monophosphate adenosine monophosphate (cGAMP). cGAMP activates the stimulator of interferon gene (STING), leading to the transport/activation of TANK-binding kinase 1 (TNK1), which phosphorylates and activates IFN regulatory factor 3 (IRF3) and NF-κB. Nuclear translocation of IRF3 induces IFN production and NF-κB activates proinflammatory signaling (Todosenko et al., 2023b).

IFN-β promoter activity, which is stimulated by adaptor molecules (Flag, RIG-I, MAVS, TBK1, IKKε, IRF3), is increased under conditions of PHB1 deficiency. PHB1 inhibits IRF3 signaling and subsequent IFN-β production through the (competitive) interaction (co-precipitation) of the PHB core domain of the PHB1 protein with the NLS region of the importins (α1, α5), thereby restricting nuclear import of IRF3 (Zou et al., 2023). In addition, binding sites for the transcription factors STAT1 and P53, which are involved in the expression of IFN-β, have been identified in the promoter of the PHB1 gene (Begitt et al., 2014).

A key role in triggering the STING-dependent IFN-1 response has been demonstrated for the regulatory and basic cristae architecture/phospholipid complexes - OPA1, MICOS (MIC19, MIC60), SAM (MTX2, SAMM50), MIB (DNAJC11), F1F0-ATP synthase, cardiolipin (PGS1, PTPMT1), PHB, ATAD3A, YME1L1 (He et al., 2022). This suggests a mechanism that combines the disorganization of mitochondrial cristae, the release of mitochondrial content into the cytoplasm and the development of an inflammatory response. Inflammation caused by cytoplasmic mtDNA via activation of the cGAS-STING pathway is a factor in aging and metabolic syndrome associated with obesity (Li and Chen, 2018; Todosenko et al., 2023b). Thus, protecting the architecture and integrity of mitochondrial cristae could prevent the development of sterile inflammation observed in metabolic disorders.

#### 3.1.2 NLRP3

Recognition of mtDNA occurs with the help of the cytoplasmic multiprotein complex of the innate immune system - the NLRP3 inflammasome, whose activation is mediated by NF-kB and the subsequent interaction of the specific and general adapter protein (ASC), which transmits a signal to trigger the cleavage of caspase-1 and the production of IL-1β and IL-18 observed in metabolic syndrome (Wu et al., 2020; Todosenko et al., 2023b).

PHB2 is involved in regulating the inflammatory response to cytosolic and extracellular mtDNA by blocking the activation of NLRP3, ASC and caspase 1 in parallel with the reduction of IL-1β and IL-18 (Xu Y. et al., 2019).

##### 3.1.2.1 Nucleus

PHBs are localized in the nucleus and interact directly/indirectly with various transcription factors, including the estrogen receptor (PHB1/2), the E2F protein family (PHB1), p53 (PHB1), MEF2 (PHB2), MyoD (PHB2), STAT3 (PHB1), orphan nuclear hormone receptors (COUP-TFI/II; PHB2), PPARγ (PHB2) (Bavelloni et al., 2015; Peng et al., 2015) (Figure 2).

The accumulation of PHB1 proteins in the nucleus is associated with impaired cell division and cell cycle arrest (Liu et al., 2012). The functions of PHB1 can be defined as follows: 1) repression of gene transcription; 2) p53-mediated transcriptional induction; 3) Ras signaling (via interaction with Raf1) (Qi et al., 2023). Low expression of PHB1 leads to activation of the transcription factors NF-κB and AP-1, which induces the expression of IL-8. However, PHB1 is able to bind to JUN, indicating a functional potential as an AP-1 co-repressor (Yang et al., 2019).

PHB2 has similar intranuclear functions which are complemented by transcriptional regulation of estrogen receptor, myocyte enhancer factor 2 (MEF2), myoblast determination protein 1 (MyoD), and peroxisome proliferator-activated receptor (PPARγ) (Thuaud et al., 2013; Qi et al., 2023). Mechanistically, PHB2 forms intranuclear complexes with MyoD and MEF2 and represses their transcription. A direct binding of PHB2 to MyoD and an indirect interaction with MEF2 was observed.

The phb2 promoter is thought to contain binding regions for more than 130 different transcription factors (8, 87). These transcription factors include negative regulators of phb2 expression (ERα) and factors that induce phb2 expression (Sp1) (Bavelloni et al., 2015). PHB2 inhibits COUP-TF1/II, an orphan nuclear hormone receptor (NHR), by recruiting histone deacetylases (HDACs) (Kurtev et al., 2004). PHB2 interacts with HDAC1 and HDAC5 and also recruits HDACs to transcription sites and represses nuclear receptor transcription (Qi et al., 2023) (Figure 1).

PHB2 contacts the cotranscriptional PPARγ activator PGC-1α and inhibits PPARγ transcription (Thuaud et al., 2013). PPARγ regulates the adipogenesis of cells, whereby the differentiation of adipocytes is controlled by PHB (Pant et al., 2020). Insulin, leptin and PPARγ interact with insulin and C/EBP contact sites in the promoter of the PHB gene and induce PHB protein expression, activating adipogenesis in 3T3-L1 cultures *in vitro*. The functions of PHB associated with membrane localization play an important role in maintaining adipocyte viability in obesity (Kang et al., 2013; Ande et al., 2014; Gao Z. et al., 2021). PHB functions as a target protein for Akt (Jiang et al., 2015; Ande et al., 2017). PHB may have a regulatory effect on the activation of the PI3K/Akt and Raf/MAP/ERK signaling cascades and regulates the phosphorylation process (Ande et al., 2012) (Figure 1).

In addition, sexual dimorphism is associated with PHB1 regulation, obesity traits (Xu Y. X. Z. et al., 2019) and immune response (Ande et al., 2016; Nguyen et al., 2016). A link has been found between the increase in sex hormones during puberty and PHB-mediated regulation of adipose tissue development. A certain concentration of steroid hormones is necessary for the development of obesity in Mito-Ob mice, and gonadectomy prevents weight gain (Xu et al., 2018).

Thus, in the nucleus, global proteins and specific transcription factors interact with PHB2 via protein-protein connections, including the cAMP-dependent transcription factor (ATF-2), β-catenin, COUP-TF1, COUP-TF2, the estrogen receptor (ER-α), IL-3 enhancer binding factor (ILF-3), MEF2A, MYOD1, RUNX3, TF-E3 (Havugimana et al., 2012; Lau et al., 2012). In addition, PHBs interact with DNA-modifying enzyme proteins: Histone deacelases (HDAC1, HDAC2, HDAC3, HDAC5), breast cancer antigen (BRCA-1), cyclin-dependent kinase (CDK-2), Polycomb-related proteins PRC2, complex EED-EZH2, DNA repair-related enzymes and cell cycle-related proteins. RNA-binding proteins required for RNA processing (ATP-dependent RNA helicase DDX20), stability (epiplakin EPPK1), the protein expressed by basophilic leukemia (Bles03) and transport (Staufen) are also bound by PHB2 in the nucleus (Bavelloni et al., 2015).

### 3.2 Possible involvement of PHBs in signaling pathways related to inflammation and obesity

**FIGURE 3:**
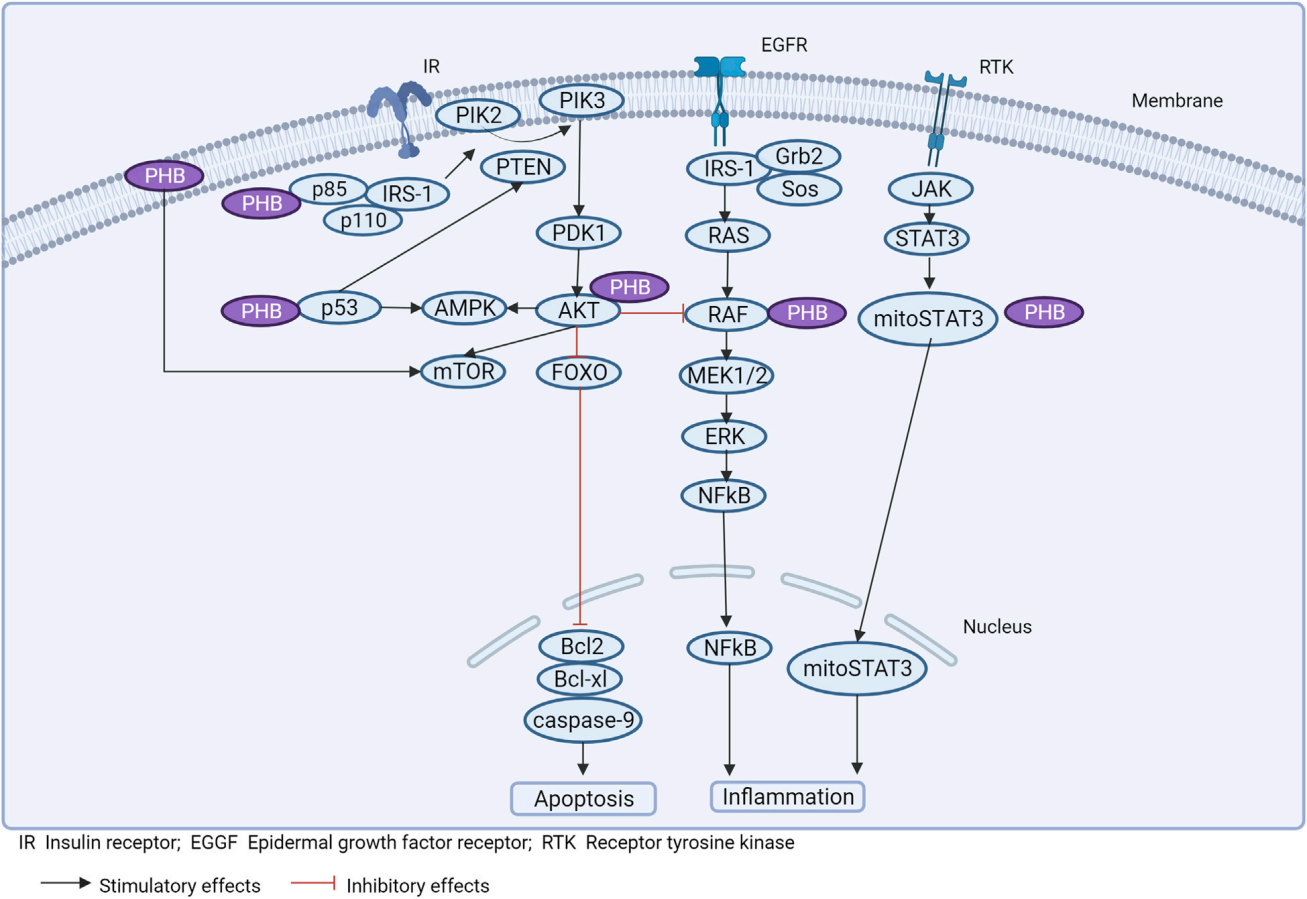
Prohibitins and signaling pathways associated with inflammation and obesity.

Experimental studies on the functional properties of PHBs in different pathologies have mainly been performed on animals or cell cultures, which makes the interpretation of the data obtained difficult. At the same time, there are only a limited number of studies aimed at determining the role of PHBs in the pathogenesis of obesity (and metabolic syndrome) (Theiss and Sitaraman, 2011). However, in conjunction with deciphering the targets with which PHBs interact, and based on the molecular genetic results described, it is possible to determine the potential role of PHBs in obesity-related signaling pathways (Figure 3).

#### 3.2.1 WNT for obesity

There are three signaling pathways associated with Wnt: the canonical pathway, the non-canonical cell polarity pathway, and the non-canonical calcium signaling pathway. The canonical pathway is mediated by the binding of Wnt ligands to the Frizzled receptor and the LRP-5/6 coreceptors on the cell surface and leads to the translocation of cytoplasmic β-catenin to the nucleus and the activation of transcription factors (TCF4/7, LEF) (Abou Ziki and Mani, 2019). In the inactivated state, β-catenin interacts with the destruction complex (APC, AXIN, GSK3β, CK1α), which phosphorylates β-catenin and ensures its proteasomal degradation (Alula et al., 2021). In the non-canonical cell polarity pathway, Wnt ligands interact with the Frizzled receptor and RYK/ROR co-receptors to activate Rho/Rac signaling. The non-canonical calcium signaling pathway is triggered by the binding of Wnt ligands to Frizzled receptors and the activation of phospholipase C (PLC), resulting in intracellular calcium release (Abou Ziki and Mani, 2019). These Wnt signaling pathways are mutually regulated; upon phosphorylation of LRP-5/6, canonical signaling is activated and non-canonical signaling is inhibited (Abou Ziki and Mani, 2019).

Obesity and metabolic diseases are associated with impaired Wnt signaling, whose activators are fatty acids and glucose, leading to adipocyte hyperplasia and the development of local and systemic inflammation (Anagnostou and Shepherd, 2008; Liu et al., 2016; Zuriaga et al., 2017; Chen and Wang, 2018; Abou Ziki and Mani, 2019). LRP6 mutations (R611C, R473Q, R360H, N433S) are associated with increased body fat, IR and decreased insulin receptor expression. Concomitantly, non-canonical Wnt signaling is increased and TCF7L2 expression is inhibited, leading to activation of the Mtorc1 pathway (also in an AMPK-dependent manner), serine phosphorylation of IRS1, promotion of NAFLD development, and activation of the TGF-β pathway.

AMP-activated protein kinase (AMPK) is an important sensor of cellular energy through direct interaction with adenine nucleotides (ATP, ADP, AMP). A reduced energy balance leads to activation of AMPK and regulation of cellular adaptation by downstream messenger molecules and gene transcription (Trefts and Shaw, 2021). However, a high-fat diet inhibits AMPK phosphorylation by inducing mTOR phosphorylation (Li et al., 2022a).

In obesity, GSK3β expression is increased in the hypothalamus, which exacerbates weight gain in HFD and blocks insulin signaling, leading to leptinergic resistance. AKT is able to inhibit GSK3 by triggering leptin/insulin signaling via PI3K (Li et al., 2022a). A polymorphism in the TCF7L2 gene that enhances its transcription is a risk factor for the development of type 2 diabetes (Obianom et al., 2019).

AXIN has been found to be involved in lipid metabolism. Knockout of AXIN (inactivation of AMPK-LKB1) in the liver of mice led to fatty degeneration (lipid accumulation) (Zhang et al., 2013). Liver-specific knockout of β-catenin had an antifibrotic effect (in mice) (Zhang et al., 2013) and increased insulin sensitivity y (Popov et al., 2016).

High levels of fatty acids promote Wnt-induced β-catenin stability, and PLIN2 is a lipid droplet-associated protein and GSK3 that affects Wnt/β-catenin signaling. The interaction between β-catenin and PLIN2 has been demonstrated in cell cultures (3T3-L1, HEK293, THP-1) under obesity-modulating conditions (Liu et al., 2016).

The master regulator of the cell cycle is the transcription factor E2F1, which activates the MAP kinase signaling pathway (ASK1-MKK4-p38MAPK/JUN) in the fat cells of visceral adipose tissue (steatosis in the liver) and thus triggers the inflammation/dysfunction of fat cells (and IR) in obesity. High expression of E2F1 induces TRAIL (TNFSF10), which upregulates TL1A (TNFSF15) in adipose tissue T lymphocytes and stimulates the formation of proinflammatory macrophages (M1) that accumulate lipids, leading to IR, inflammation (IL-6) and increased E2F1 expression in adipocytes (Maixner et al., 2020).

The retinoblastoma pocket protein (Rb) is activated by DNA damage and inactivates E2F1. Phosphorylation of Rb by cyclin-dependent kinase (CDK4) causes the release of E2F1 (dissociation) and transcriptional activation of target genes (from G1 to S phase) (Dubrez, 2017).

HFD leads to the development of leptin resistance in the brain and activation of AMPK in anorectic hypothalamic neurons (POMC), triggering RB1 phosphorylation and E2F1 activation (Lu et al., 2013; Lyu et al., 2020). At the same time, the AMPKα2 subunit is the target gene for E2F1 in REF53 cells (Namba et al., 1994), indicating the likely formation of a vicious cycle of HFD-AMPK-Rb1-E2F1-AMPK in hypothalamic POMC neurons (Lu et al., 2013).

Literature data suggest an important regulatory role of PHBs in the (WNT)/β-catenin signaling cascade. Overexpression of PHB1 was found to alter (WNT)/β-catenin activation only after neoplastic progression. The rate-limiting component of the β-catenin degradation complex, AXIN1, which functions as a tumor suppressor protein, has a putative PHB1-binding domain in the promoter region (Jackson et al., 2020). In the nucleus, PHB1 is a potent inducer of AXIN1 expression by inhibiting (WNT)/β-catenin signaling (Alula et al., 2021). PHB1 is a direct target gene of WNT via the TCF4 site in the PHB1 promoter in leukemia cells (Kim et al., 2017). Overexpression of PHB1 in adenomas decreased the expression of WNT target genes (Mmp7, Ccnd1, Tcf4), blocked cell proliferation and increased cell apoptosis (Alula et al., 2021). In addition, PHB1 is able to block the WNT/β-catenin signaling pathway (4) in an E2F1-dependent manner by inhibiting the activation of Wnt10a and Wnt9a promoters (in hepatocellular carcinoma cells) (Mavila et al., 2018; Barbier-Torres and Lu, 2020). This effect is achieved by the formation of the PHB1-RNF2 protein complex, which is recruited to the E2F1 response promoter and regulates its transcriptional activity. PHB1 also regulates E2F1 transcriptional activity via the p16-CDK4-Rb signaling pathway, thereby decreasing p16 activity and increasing E2F1 transcription (Choi et al., 2008).

In this case, PHB1/PHB2 forms a complex with AMPK and blocks signal transduction. Disruption of the interaction of PHB1 with AMPK induced by RX-375 resulted in activation of AMPK, reducing inflammation and hepatic steatosis in obese mice (Kanagaki et al., 2023) (Figure 3).

#### 3.2.2 P53 for obesity

In obesity and components of the metabolic syndrome (NAFLD) (Bonnet et al., 2022), p53 expression (and activation of the canonical pathway) increases in cells, associated with the aging process of adipocytes and the development of insulin resistance (Franck et al., 2013; Kastenhuber and Lowe, 2017; Krstic et al., 2018). Genetic hyperphagia (Ay mice) resulted in increased protein levels of p53, ROS, active β-galactosidase, pro-inflammatory cytokines and chemokines, leading to IR in epididymal adipose tissue (Krstic et al., 2018) (. P53 emerges as a key factor in adipose tissue development under chronic HFD-induced stress (Porteiro et al., 2018).

Additionally, p53 induction in visceral adipose tissue activates the semaphorin 3E (Sema3e) target gene and triggers the p53-semaphorin 3E (Sema3e)-plexin D1 signaling cascade, leading to macrophage recruitment, oxidative stress, and inflammation (Shimizu et al., 2013; Vergoni et al., 2016; Ogrodnik et al., 2017; Krstic et al., 2018).

At the same time, reprogramming of adipocyte (glucose) metabolism with changes in diet is associated with activation of the non-canonical p53 pathway, associated with the regulation of the p53 target gene - lysosomal acid lipase (LAL), responsible for the breakdown of triglycerides and cholesteryl esters in lysosomes, which has a similar sequence in the promoter region p53 response element (Wang et al., 2020). This pathway involves AMPK, under the influence of which phosphorylation/activation of p53 transcription occurs (in response to changes in the ATP/AMP ratio) and subsequent induction of LAL (Wang et al., 2020). However, in obesity, activation of the non-canonical p53 pathway is observed without changing the ATP/AMP ratio, probably with the participation of other factors regulating LAL transcription.

An important role of PHB in the regulation of p53 expression under normal conditions and pathologies has been noted (Germain, 2011). PHB1 directly binds to the p53 inducible gene 3 (PIG3) promoter motif (TGTCC), promoting p53-dependent cell apoptosis (Guan et al., 2014).

PHB interacts with p53, enhancing transcriptional activity (preventing p53 denaturation), mediating an antitumor effect. PHB binds to phospho-STAT3 and induces a decrease in the expression of Bcl-xL, Bcl-2 (Kathiria et al., 2012) (Figure 3).

#### 3.2.3 NF-kB, MAPK

The inflammatory response mediated by cells of the immune system plays a key role in obesity. Activation of MAPK and NF-kB signaling pathways is observed in various organs and tissues associated with obesity (Gao X. et al., 2021).

In monocytes, phosphorylation of components of the MAPK/NF-kB signaling cascades (MAPK/JNK, ERK1/2, p65NF-kB) is observed in a background of TNFα-mediated low-grade systemic inflammation, which promotes the chronicity of inflammation involving ASCL1 (Al-Roub et al., 2023).

Furthermore, free saturated fatty acids induce the expression of S100A9 on macrophages, activate TLR4-NF-kB signaling and modulate the formation of proinflammatory macrophages (M1) (Franz et al., 2022).

Inflammation can induce mutational variability in PHB and disrupt signaling associated with ERK and STAT6 (Xu et al., 2022). Membrane PHB is involved in the activation of Ras/Raf-1 kinases and MAPK isoforms ERK1/2 under the influence of external stimuli (Mishra et al., 2010). PHB physically binds to Raf and activates Ras-induced Raf/MAP/ERK signaling (upon EGF stimulation), which regulates proliferation, differentiation, cell apoptosis and inflammation (Mishra et al., 2010).

#### 3.2.4 MYC-cGAS-STING

MYC regulates metabolic homeostasis at the systemic level. Induction and amplification of c-MYC transcription is observed in response to consumption of a high-fat diet (Atala, 2020). The progression of cellular senescence is associated with overexpression of c-MYC, which causes DNA damage and increases ROS levels. c-MYC induces DNA damage and increases ROS levels by activating the cGAS-STING signaling pathway (Ning et al., 2020), as observed in obesity and metabolic syndrome (Todosenko et al., 2023b). The promoter of the STING gene contains a c-MYC binding site and regulates STING expression (Hofmann et al., 2015).

Heteredimerization of PHB1 with transcription factors (MAX) suppresses the activity of the promoters of the E-Box, MYC, MAFG and c-MAF genes (Fan et al., 2017).

Lowering MYC levels in obese mice had beneficial effects on HFD-induced obesity, IR, steatosis and steatohepatitis (Luo et al., 2021). When c-MYC binds to the STING promoter, STING transcription is inhibited and cGAS activity is increased, leading to a decrease in the secretion of proinflammatory mediators (Sundaram et al., 2022).

It is likely that PHB has a modulatory effect on the regulation of the inflammatory response in several ways, by modulating mitochondrial structural architecture and cristae integrity, preventing mtDNA shedding and altering the transcription of nuclear genes by interacting with their promoter regions, which reduces inflammation in obesity (Figure 3).

## 4 Therapeutic strategies

### 4.1 Drugs that target PHB

Molecular agents targeting PHB are in active development and may form the basis of new therapeutically feasible treatments for immune and metabolic diseases (Mishra and Nyomba, 2017).

A peptide motif (sequence CKGGRAKDC), which is present in white adipose vessels and has been shown to have a proapoptotic effect, triggers the degradation of white fat when bound to PHB. Resorption of the resulting white adipose tissue and the normalization of metabolism led to the disappearance of obesity with no apparent side effects (in experimental animals) (Kolonin et al., 2004). Adipotides targeting KGGRAKD to endothelial PHB reduce adipose tissue endothelial cells and reverse obesity in animal models (Gao et al., 2022).

Clinical trials with adipotides have been conducted in humans with obesity and cancer. Since PHBs are expressed on adipocytes and endothelial cells, the efficacy of adipotide may be due to its transendothelial transport and access to adipocytes (Salameh et al., 2016). Thus, the action of CKGGRAKDC on the PHB receptor complex in white adipocytes promoted apoptosis of white adipose endothelial cells and thus prevented obesity (Hossen et al., 2013; Barbier-Torres and Lu, 2020).

Small molecules affecting PHB function have been found to block the utilization of long-chain fatty acids and activate glucose utilization in adipocytes (Gao Z. et al., 2021, 2022). PHB ligands with different pharmacological properties include FL3, Mel6, Mel41, JI130 and fluoroisoline. Furthermore, FL3, in addition to PHB, interacts directly with helicase and induces potent anticancer effects (Basmadjian et al., 2013; Boussemart et al., 2014).

Fluoroisoline is a potent cytotoxic PHB ligand that causes mitochondrial fragmentation and inhibits PHB-dependent activation of the C-RAF signaling cascade. In addition, fluoroisoline inhibits mTORC1 signaling and affects S6K and eIF4E-BP1. Fluoroisoline also induces calcium influx leading to phosphorylation of key factors that regulate protein synthesis (eIF2, eEF2). However, this effect of fluorisoline only occurs in in vitro experiments.

Small molecules targeting PHB have shown promising effects against cancer, neurodegenerative, metabolic and inflammatory diseases.

The capsular polysaccharide Vi (Vi) from *Salmonella typhi* has been shown to suppress the immune response by interacting with the PHB membrane and leading to suppression of ERK activity and IL-8 secretion in human intestinal epithelial cells. Vi blocks the GTPases Rac1, Cdc42, NF-kB signaling, ERK and actin cytoskeleton remodeling in monocytes and T cells, thereby suppressing the immune response (Santhanam et al., 2014). Vi also blocks C-RAF activation in PHB1-positive Th17 lymphocytes (Buehler et al., 2018).

Spirooxindoles are also a class of ligands for PHB that exert a protective effect against doxorubicin (cardiomyocytes) (Elderwish et al., 2020). Their mechanism of action is related to STAT3 phosphorylation and its translocation to the mitochondria.

The arginine derivative PDD005 is a PHB ligand that corrects age-related cognitive impairment in mice. The compound inhibits neuroinflammation and reverses deficits in neurogenesis (Guyot et al., 2020).

In addition, substances from the flavogline class (Sylvestrol, IMD-019064, FL3), aurilides, melanogenins, capsaicins, sulfonylamidine derivatives targeting PHB (Thuaud et al., 2013) have anti-inflammatory and anti-cancer effects. In particular, the synthetic ligand PHB2–FL3 inhibited the mitochondrial recruitment of PRKN and the accumulation of PINK1 in depolarized mitochondria, thus blocking PINK1/PRKN mitophagy (Yan et al., 2020; Belser and Walker, 2021).

### 4.2 Targeted nanotherapy against PHB

Potentially, peptides that interact with cell receptors can be used to deliver specialized molecules that neutralize the transport function of the PHB/ANX2/CD36 complex and redirect signals in white adipose tissue, hepatocytes and other cells for the treatment of obesity, hepatosteatosis, type 2 diabetes mellitus and metabolic disorders (Rupert and Kolonin, 2022).

The specificity and efficacy of conventional anti-obesity drugs can be improved by the development of molecular drug carriers based on biocompatible polymers (polymer conjugates, hydrogels, microneedles, nanoparticles, liposomes) with controlled release of the substance. The basis of modern molecular structures for the transfer of therapeutic components is the process of drug conjugation with a polymer chain, encapsulation or introduction into a carrier matrix.

An example of the effective use of an oral PEGylated (polyethylene glycol bound to a drug substance) anti-obesity drug is the drug semaglutide (Aroda et al., 2019; Pieber et al., 2019; Overgaard et al., 2021), which has undergone Phase 3 clinical trials and is FDA approved.

There are currently no approved drugs based on nanostructures for obesity (Sibuyi et al., 2019). At the same time, the development of nanotherapeutic approaches for the treatment of obesity targets white adipose tissue cells, including inhibition of angiogenesis (Hossen et al., 2014), conversion to brown adipose tissue by induction of mitochondrial biogenesis (Zhang et al., 2017), and photothermal lipolysis of white adipose tissue (Lee et al., 2017). Since PHB serves as a biomarker for white adipose tissue vasculature and angiogenesis and adipogenesis are linked (Cao, 2014), PHB is used to deliver cytotoxic agents to vascular endothelial cells to inhibit angiogenesis and the development of adiposity (Sibuyi et al., 2018).

A potential method for targeted drug delivery based on metallic and/or biodegradable nanoparticles containing apoapoptotic peptides is to attach to their surface a sequence that targets adipose tissue - a lipid peptide that interacts with PHB. Metallic nanoparticles are able to directly contact mitochondria, and biodegradable polymer nanoparticles gradually release their content, triggering the release of cytochrome C and the activation of caspases, leading to cellular PHB-dependent apoptosis (Hossen et al., 2012, 2014).

In mice (Jiang et al., 2017) and obese pigs (Huang et al., 2020), the conversion of white to brown adipocytes was achieved by enhanced mitochondrial biogenesis by targeting dibenzazepine (an antidepressant) to white adipose tissue using polylactide coglycoside nanoparticles. This nanocarrier resulted in increased energy expenditure and an improved metabolic phenotype (Jiang et al., 2017; Huang et al., 2020). Rosiglitazone (a class of thiazolidinediones) encapsulated in polymeric and glucose-sensitive dextran nanoparticles (Xue et al., 2016; Zhang et al., 2017) targeting PHB-positive adipose tissue endothelial cells had a similar effect. A non-invasive patch containing dextrin nanoparticles (rosiglitazone, glucose oxidase, catalase) placed in silicone tips of hyaluronic acid microneedles and targeting subcutaneous fat cells (Zhang et al., 2017) was also tested in obese mice. Patches containing rosiglitazone resulted in significant weight loss and localized darkening of adipose tissue in mice (Zhang et al., 2017).

Gold nanospheres have also been developed that non-invasively penetrate three layers of skin, reach the target tissue and destroy cells (necrosis, apoptosis) by photothermal activation (conversion of near-infrared light into heat after laser excitation). Hyaluronic acid and fat peptides were attached to these nanospheres, which, when applied topically in the peritoneal cavity of obese mice, establish contact with PHB-expressing cells (Lee et al., 2017).

Based on the above, it is possible to consider PHB as a potential molecular target for metabolic disorders. However, the tissue-specific action of PHB and its localization (membrane, mitochondria, nucleus) need to be further and more thoroughly investigated to understand its role in the pathogenesis of metabolic syndrome components in detail and to develop effective molecular genetic constructs with high therapeutic potential.

## 5 Conclusion

The mechanism by which obesity and metabolic disorders develop with overeating involves multiple cellular, molecular and genetic aspects that contribute to the complexity of interpretation and the discovery of effective therapeutic targets. Hormone levels are implicated in the development of metabolic diseases, highlighting the need for a personalized approach in the treatment of these diseases.

Prohibitins are a key element in the regulation of cellular homeostasis, in particular by modulating the response at different levels: Nucleus, mitochondria, membrane. Their localization and interaction with different proteins, hormones, transcription and nuclear factors and mtDNA indicate the globality and complexity of their pleiotropic properties, which remain to be explored. A more detailed deciphering of the cellular metabolism associated with prohibitins under normal conditions and in different metabolic diseases will allow to understand the exact role of prohibitins in the signaling cascades of PI3K/Akt, Raf/MAP/ERK, STAT3, p53 and others and to fathom their mutual influence. An interesting fact is the circulation of PHB in the bloodstream and the effect on systemic functions of the body. It has been reported that PHB1 is present in human serum as part of lipid droplets, where PHB1 interacts with complement protein C3 to trigger an innate immune response (Thuaud et al., 2013). In addition, some cancers (colorectal cancer) are associated with a marked increase in PHB1 and PHB2 levels in blood serum (Mengwasser et al., 2004). Investigation of this phenomenon may open up the possibility of non-invasive detection of PHB and its potential as a predictor and marker for various pathologies. Of particular interest for the assessment of the degree of inflammation is the fact that PHB1/PHB2 is present in free form in the bloodstream and has a regulatory effect at the systemic level (Mishra et al., 2006).

Investigating the role of prohibitins in the molecular and cellular interactions between two key players in the pathogenesis of obesity–adipocytes and macrophages, which form the basis of the metainflammatory response, offers a valuable research perspective. The study of the fine intercellular communication and molecular cascades triggered in these cells will allow us to propose new therapeutic strategies to eliminate persistent inflammation, taking into account new molecular genetic approaches targeting the activation/inactivation of prohibitins.

However, given the existing molecular genetic developments targeting PHB and the investigation of their therapeutic potential, we can confidently consider this area of medicine as promising.
